# New Andrastin-Type Meroterpenoids from the Marine-Derived Fungus *Penicillium* sp.

**DOI:** 10.3390/md19040189

**Published:** 2021-03-27

**Authors:** Jinwei Ren, Ruiyun Huo, Gaoran Liu, Ling Liu

**Affiliations:** 1 State Key Laboratory of Mycology, Institute of Microbiology, Chinese Academy of Sciences, Beijing 100101, China; renjw@im.ac.cn (J.R.); ruiyunhuo@163.com (R.H.); liugaoran@163.com (G.L.); 2 College of Life Sciences, University of Chinese Academy of Sciences, Beijing 100039, China

**Keywords:** marine-derived fungus, secondary metabolites, meroterpenoids, absolute configurations, cytotoxicity

## Abstract

Three new andrastin-type meroterpenoids penimeroterpenoids A–C (**1**–**3**) together with two known analogs (**4** and **5**) were isolated from the cultures of the marine-derived *Penicillium* species (sp.). The structures of the new compounds were elucidated on the basis of 1- and 2-dimensional (1D/2D) Nuclear Magnetic Resonance (NMR) spectroscopic and mass spectrometric analysis. The absolute configurations of **1**–**3** were determined by comparison of experimental and calculated electronic circular dichroism (ECD) spectra. Compound **1** showed moderate cytotoxicity against A549, HCT116, and SW480 cell lines.

## 1. Introduction

Meroterpenoids are hybrid natural products with partial structure fragments derived from terpenoids [1,2]. Based on biosynthetic origins, meroterpenoids can be sorted into polyketide-terpenoids and nonpolyketide-terpenoids [1,2]. The andrastin-type meroterpenoids derived from 5-dimethylorsellinic acid (DMOA) and farnesyl diphosphate (FPP) via a mixed polyketide-terpenoid pathway are characterized by a five-methyl substituted ent-5*α*,14*β*-androstane skeleton (6,6,6,5-tetracarbocyclic skeleton) [3,4]. Since citreohybridones A and B were isolated in 1991, over 30 analogues have been isolated and characterized [5,6,7,8,9,10,11,12,13,14]. Many andrastin-type meroterpenoids have shown potent cyotoxic, antifeedant, and insecticidal activities [4,8,10]. Andrastin-type meroterpenoids have attracted much attention from synthetic chemists due to their structural and biological diversity [15,16,17]. In recent years, the biosynthetic pathways involved in andrastin-type meroterpenoid production have been well-elucidated [4,14,18,19].

**Figure 1:**
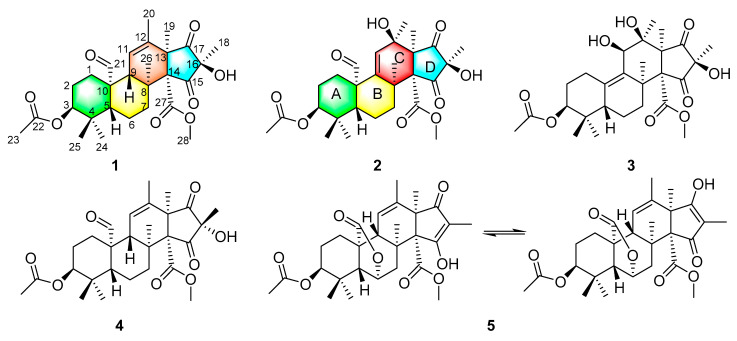
Structures of compounds **1**–**5.**

Marine-derived fungi, living under extreme environmental conditions such as high salinity, intensely high pressure, absence of sunlight, and deficiency of nutrients, are considered to be a new reservoir of structurally diverse and biologically active metabolites for drug discovery [20,21]. In our ongoing search for new bioactive secondary metabolites from marine-derived fungi, the fungus *Penicillium* sp. (A18), isolated from a deep-water sediment sample that was collected at a depth of 5115 m in the East Pacific, was selelected for chemical investigations. As a result, three new andrastin-type meroterpenoids, which have been named penimeroterpenoids A–C (**1**–**3**), together with two known compounds, andrastone E (**4**) [22] and citreohybridonol (**5**) [11] (Figure 1), were isolated and identified from the culture extract of the fungus. Their structures were established by a detailed interpretation of 1D/2D NMR spectroscopic and mass spectrometric data, and the absolute configurations of **1**–**3** were determined by electronic circular dichroism (ECD) calculations. All of these compounds were evaluated for cytotoxicity against a panel of six human tumor cell lines. Herein, the details of the isolation, structure elucidation, and biological activity of these compounds (Figure 1) are described.

## 2. Results

**Figure 2:**
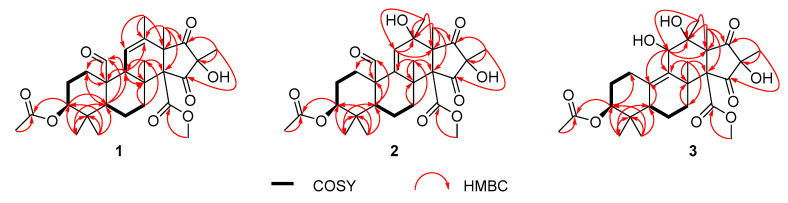
Key correlation spectroscopy (COSY) correlations and heteronuclear multiple bond correlations (HMBC) of **1**–**3**.

Penimeroterpenoid A (**1**) was obtained as a colorless oil. Its molecular formula C_28_H_38_O_8_ was established by high-resolution electrospray ionisation mass spectrometry (HRESIMS) analysis (*m/z* 525.2454 [M + Na]^+^), indicating ten degrees of unsaturation. The infrared (IR) spectrum showed the presence of hydroxy (3445 cm^−1^) and carbonyl (1752 cm^−1^) groups. Analysis of its NMR data (Table 1) revealed the presence of eight methyl groups, four methylenes, three methines (including one oxymethine), six sp^3^ quarternary carbons (one oxygenated), one trisubstituted olefin unit, two carboxylic carbons (*δ*_C_ 167.3, 170.7), one aldehyde group (*δ*_C_ 204.5; *δ*_H_ 10.1), and two ketone carbons (*δ*_C_ 210.6, 206.8, respectively). These data accounted for all ^1^H and ^13^C NMR resonances except for one exchangeable proton, suggesting that **1** was a tetracyclic compound. Analysis of the ^1^H-^1^H correlation spectroscopy (COSY) NMR data (Figure 2) led to the identification of three isolated spin-systems of C-1–C-2–C-3, C-5–C-6–C-7, and C-9–C-11. Heteronuclear multiple bond correlations (HMBC) from H_2_-1 to C-10, H-3 to C-5, H-5 to C-1, C-4, C-10, C-24, and C-25, and from the geminal methyl groups H_3_-24 and H_3_-25 to C-3, C-4, and C-5 completed the cyclohexane ring (ring A). HMBC cross-peaks from H-3 and H_3_-23 to the carboxylic carbon C-22 (*δ*_C_ 170.7) established the location of the acetyl group at C-3. Other correlations from H-5 to the aldehyde carbon C-21, and from the aldehyde proton H-21 to C-1 and C-10 indicated that C-21 was attached to C-10. While the HMBC cross-peaks from H_2_-1 and H-5 to C-9, H_2_-7 to C-8 and C-26, H-9 to C-8, C-10, C-21, and C-26, and from H_3_-26 to C-7, C-8, C-9, and C-14 permitted the completion of another cyclohexane unit (ring B) with the methyl carbon C-26 and the sp^3^ quarternary carbon C-14 (*δ*_C_ 70.6) attached at C-8. HMBC correlations from H-9 to C-12, H-11 to C-8, C-10, C-13, C-20, H_3_-19 to C-12, C-13, C-14, and C-17, and from H_3_-20 to C-11, C-12, and C-13, as well as from H_3_-18 to C-16 and two ketone carbons (C-15 and C-17: *δ*_C_ 210.6 and 206.8, respectively) permitted the completion of the tetrahydro-1*H*-indene-1,3(2*H*)-dione moiety (rings C and D), fused with the cyclohexane ring B at C-8 and C-9. In addition, HMBC correlations from H_3_-28 to the carboxylic carbon C-27 (*δ*_C_ 167.3) located the methoxy group at C-27. The exchangeable proton was located at C-16 by default, an identification supported by the chemical shift value for C-16 (*δ*_C_ 72.1). Thus, the planar structure of **1** was established as shown (Figure 1), and has the same planar structure as compound **4**.

**Figure 3:**
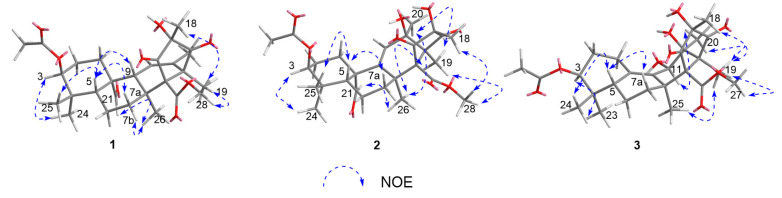
Key nuclear overhauser effect spectroscopy (NOESY) correlations of **1**–**3.**

The relative configuration of **1** was assessed by analysis of the nuclear overhauser effect spectroscopy (NOESY) correlations (Figure 3). NOESY correlations of H-5 with H-7a, H-9, and H_3_-25 indicated that these protons are all on the same side of the ring system. While NOESY correlations of H-3 with H_3_-24, H_3_-26 with H-7b and H-21, and of H_3_-28 with H_3_-19 and H_3_-18 placed these protons on the opposite side of the tetracyclic system. Futhermore, the NOESY correlation between H-9 and H_3_-18 in **1** disappeared compared to **4**, and the carborn signal for C-16 and C-18 was shifted upfield by 3.8 and 5.8 ppm, suggesting an inversion of the C-16 stereocenter. These observations led to the assignment of the relative configuration of **1,** indicating that **1** was the C-16 epimer of compound **4**.

**Figure 4:**
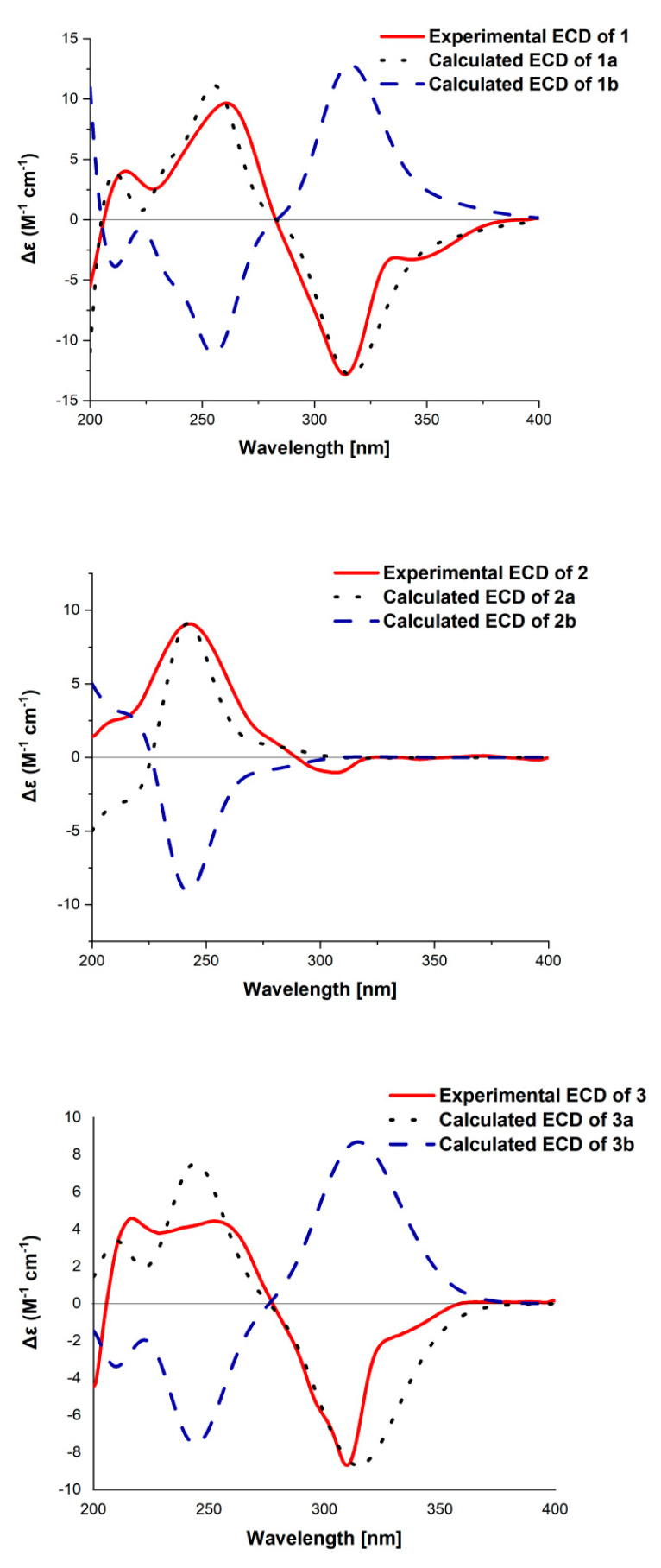
Calculated and experimental electronic circular dichroism (ECD) spectra of **1**–**3**.

The absolute configuration of **1** was assessed by comparison of the experimental and simulated ECD spectra generated by the time-dependent density functional theory (TDDFT) for two enantiomers (3*S*,5*R*,8*S*,9*R*,10*S*,13*R*,14*R*,16*R*)-**1** (**1a**) and (3*R*,5*S*,8*R*,9*S*,10*R*,13*S*,14*S*,16*S*)-**1** (**1b**). The MMFF94 conformational search and density functional theory (DFT) re-optimization at the CAM-B3LYP/6-31G(2d,p) level yielded nine lowest-energy conformers for **1a** (Figure S22). The overall calculated ECD spectra of **1a** and **1b** were then generated by Gaussian broadening (Figure 4). The experimental ECD spectrum of **1** was nearly identical to the calculated ECD spectrum for **1a**, clearly indicating the 3*S*,5*R*,8*S*,9*R*,10*S*,13*R*,14*R*,16*R* absolute configuration for **1**.

Penimeroterpenoid B (**2**) was also obtained as a colorless oil. The molecular formula was determined as C_28_H_38_O_9_ (ten degrees of unsaturation) by HRESIMS (*m/z* 541.2402 [M + Na]^+^), which is 16 mass units higher than that of **1**. The IR spectroscopy indicated the presence of hydroxy (3421 cm^–1^) and carbonyl (1757 cm^–1^) groups. Analysis of its NMR data (Table 1) revealed the presence of the same partial structure as that found in **1**, except that those corresponding to the cyclohexene ring (ring B) in **1** were different in **2**. Notably, the resonances for a methine unit (*δ*_H_/*δ*_C_ 2.19/53.5, C-9), one C-11/C-12 olefin (*δ*_H_/*δ*_C_ 5.82/126.4; 132.9), and one methyl (*δ*_H_/*δ*_C_ 1.68/18.9, C-20) in **1** were replaced by those for one C-9/C-11 olefin (*δ*_H_/*δ*_C_ 5.55/125.8; 147.9), one oxygenated sp^3^ quarternary carbon (*δ*_C_ 76.0, C-12), and one methyl group (*δ*_H_/*δ*_C_ 1.26/24.3, C-20) in the spectra of **2**, indicating that the double bond at C-11/C-12 was transferred to C-9/C-11. These observations were also confirmed by HMBC correlations (Figure 2) from H-11 to C-8, C-9, C-10, C-12, C-13, and C-20, and from H_3_-20 to C-11, C-12, and C-13. On the basis of these data, the gross structure of **2** was established as shown. Compound **2** was deduced to have the same relative configuration as **1** by a comparison of their NOESY data (Figure 3). In the NOESY spectrum, the cross-peaks of H-5 with H-7a and H_3_-25 demonstrated that these protons were cofacial and were arbitrarily assigned as *β*-orientations. Meanwhile, the NOESY correlations of H-3 with H_3_-24, H_3_-26 with H_3_-19, H_3_-20, H-21, and H_3_-28, and of H_3_-28 with H_3_-18 and H_3_-19 indicated that these groups were correspondingly assigned as *α*-orientations. The absolute configuration for **2** was also proposed by a comparison of the experimental and calculated ECD spectra for the enantiomers (3*S*,5*R*,8*S*,10*S*,12*R*,13*S*,14*R*,16*R*)-**2** (**2a**) and (3*R*,5*S*,8*R*,10*R*,12*S*,13*R*,14*S*,16*S*)-**2** (**2b**). The calculated ECD spectrum of **2a** showed a good agreement with the experimental one (Figure 4), which supported the absolute configuration being 3*S*,5*R*,8*S*,10*S*,12*R*,13*S*,14*R*,16*R*. Thus, the structure of **2** was elucidated, as depicted in Figure 1.

The molecular formula of penimeroterpenoid C (**3**) was determined to be C_27_H_38_O_9_ (nine degrees of unsaturation) by HRESIMS (*m/z* 529.2409 [M + Na]^+^), which is 12 mass units fewer than that of **2**. Interpretation of the IR and NMR spectroscopic data of **3** revealed some structural features similar to those present in **2**. The main differences were that the resonances for one sp^3^ quarternary carbon (*δ*_C_ 55.1, C-10) and one trisubstituted olefin C-9/C-11 (*δ*_H_/*δ*_C_ 5.55/125.8; 147.9) in **2** were replaced by those for one tetrasubstituted olefin C-9/C-10 (*δ*_H_/*δ*_C_ 132,7; 141.9) and one oxygenated methine (*δ*_H_/*δ*_C_ 4.75/70.8, C-11) in the NMR spectra of **3**. In addition, the resonances for the aldehyde group (*δ*_C_ 202.1; *δ*_H_ 10.1) in **2** disappeared in **3**. Such an observation was also confirmed by HMBC correlations (Figure 2) from H-5 to C-10, H-11 to C-8, C-9, C-10, C-12, and C-13, and from H_3_-25 to C-7, C-8, C-9, and C-14. Therefore, the planar structure of **1** was established as shown (Figure 1). The relative configuration of **3** was also deduced by the NOESY experiment (Figure 3). NOESY correlations of H-5 with H-7a and H_3_-24 suggested the *β*-orientation of these protons, whereas those of H-3 with H_3_-23, H-11 with H_3_-20, and of H_3_-19 with H_3_-18, H_3_-20, H_3_-25, and H_3_-27 indicated that these protons were α-oriented. The absolute configuration for **3** was further determined by a comparison of the experimental and calculated ECD spectra for the enantiomers (3*S*,5*R*,8*S*,11*R*,12*S*,13*S*,14*R*,16*R*)-**3** (**3a**) and (3*R*,5*S*,8*R*,11*S*,12*R*,13*R*,14*S*,16*S*)-**3** (**3b**). The calculated ECD spectrum of **3a** was almost consistent with the experimental one (Figure 4). Thus, the absolute configuration of **3** was assigned as 3*S*,5*R*,8*S*,11*R*,12*S*,13*S*,14*R*,16*R.*

Two known compounds **4** and **5** were identified as andrastone E (**4**) [22] and citreohybridonol (**5**) [11], respectively, by comparing their spectroscopic data with those reported previously in the literature.

Compounds **1**–**5** were tested for their cytotoxic activities against T24 (human bladder carcinoma cell line), HeLa (human cervical carcinoma cell line), MCF-7 (human breast cancer cell line), HCT116 (human colon cancer cell line), SW480 (human colon cancer cell line), and A549 (human lung carcinoma cell line). Only compound **1** showed cytotoxic to A549, HCT116, and SW480 cell lines, with IC_50_ values of 82.61 ± 3.71, 78.63 ± 2.85, and 95.54 ± 1.46 μM, respectively, whereas the corresponding positive control cisplatin showed IC_50_ values of 14.91 ± 0.28, 20.22 ± 1.29, and 27.71 ± 0.90 μM, respectively. Compounds **2**–**4** did not show detectable inhibitory effects on the cell lines tested at 100 μM.

## 3. Experimental Section

### 3.1. General Experimental Procedure

Optical rotations were measured with an Anton Paar MCP 200 Automatic Polarimeter. Infrared spectra were obtained on a Nicolet IS5 FT-IR spectrophotometer. The 1D/2D NMR spectra were collected from a Bruker Avance-500 spectrometer using solvent signal (CDCl_3_: *δ*_H_/*δ*_C_ 7.26/77.2) as a reference. Mass data were performed on an Agilent Accurate-Mass-Q-TOF LC/MS 6520 instrument. Semi-preparative HPLC separation was Agilent HPLC instrument-equipped with a diode array detector using a YMC-pack ODS-A (10 × 250 mm, 5 μm, 2 mL/min). Open column chromatography (CC) was performed on sephadex LH–20 (Amersham Biosciences, Uppsala, Sweden) and silica gel (200–300 mesh, Qingdao Marine Chemical Factory, Qingdao, China), respectively.

### 3.2. Strain and Fermentation

The strain *Penicillium* sp. was isolated from a deep-water sediment sample that was collected at a depth of 5115 m in the East Pacific (145°2′ W, 07°37′ N). The isolate was identified as *Penicillium* sp. by sequencing the internal transcribed spacer (ITS) region of the rDNA (GenBank Accession No. MW767028). *Penicillium* sp. was grown on potato dextrose agar (PDA) at 27 °C for five days, and then several pieces of agar plugs (about 0.5 × 0.5 × 0.5 cm^3^) were added into 250 mL Erlenmeyer flasks containing 50 mL of media (glucose 4 g/L; malt extract 10 g/L and yeast extract 4 g/L) at 27 °C with shaking (170 rpm) for five days to produce the seed culture. Finally, Erlenmeyer flasks (500 mL) containing 80 g of rice and 120 mL of distilled H_2_O and 4.0 mL seed culture incubated at 25 °C for 30 days.

### 3.3. Extraction and Isolation

The rice fermentation material was extracted repeatedly with EtOAc (3 × 4.0 L), and the organic phases were evaporated to afford an extract (21.0 g), which was applied to silica gel CC, eluted with a petroleum ether (PE)/acetone gradient system to generate ten fractions (Fr. 1–10). Fr. 10 (1.0 g) was fractionated by normal pressure silica gel CC with PE/EtOAc (from 15:1 to 0:1) to generate six subfractions (Fr. 10–1 to Fr. 10–6). Fr. 10–1 (103 mg) was separated by octadecylsilanized (ODS) CC (20–100%, MeOH–H_2_O) to obtain six subfractions (Fr. 10–1–1 to Fr. 10–1–6). Fr. 10–1–3 was further purified by reversed-phase (RP) high performance liquid chromatography (HPLC; 42–70% MeCN/H_2_O for 45 min, 70–100% MeCN/H_2_O for 15 min; 2.0 mL/min) to obatin compounds **1** (*t*_R_ 39.0 min; 5.2 mg), **2** (*t*_R_ 33.9 min; 2.0 mg), **3** (*t*_R_ 48.2 min; 2.1 mg), and **4** (*t*_R_ 52.1 min; 2.8 mg). Fr. 8 (745 mg) was separated by an open ODS CC, which was eluted with MeOH/H_2_O (20–100%) to obtain nine subfractions (Fr. 8–1 to Fr. 8–9). Fr. 8–8 was further subjected to RP-HPLC (45–60% MeCN/H_2_O for 40 min; 2.0 mL/min) to yield compound **5** (*t*_R_ 27.8 min; 3.8 mg).

Penimeroterpenoid A (**1**): Colorless oil; [*α*${]}_{D}^{25}$ –79 (*c* 0.1, MeOH); UV (MeOH) *λ*_max_ (log *ε*): 206.0 (1.72), 310 (0.75) nm; CD (*c* 3.0 × 10^–3^ M, MeOH) *λ*max (Δ*ε*) 314 (–12.8), 267 (+9.6) nm, 216 (+4.0) nm; IR (neat) *ν*_max_ (cm^−1^): 3445, 2954, 1752, 1444, 1375, 1245, 1114. ^1^H and ^13^C NMR data, Table 1; HRESIMS *m/z* 525.2454 [M + Na]^+^ (calcd for C_28_H_38_O_8_Na, 525.2459).

Penimeroterpenoid B (**2**): Colorless oil; [*α*${]}_{D}^{25}$ +24 (*c* 0.1, MeOH); UV (MeOH) *λ*_max_ (log *ε*): 208.0 (1.73), 243.0 (1.76) nm; CD (*c* 3.0 × 10^–3^ M, MeOH) *λ*max (Δ*ε*) 243 (+9.1) nm; IR (neat) *ν*_max_ (cm^−1^): 3421, 2954, 1757, 1446, 1390, 1245, 1036. ^1^H and ^13^C NMR data, Table 1; HRESIMS *m/z* 541.2402 [M + Na]^+^ (calcd for C_28_H_38_O_9_Na, 541.2408).

Penimeroterpenoid C (**3**): Colorless oil; [*α*${]}_{D}^{25}$ –97 (*c* 0.1, MeOH); UV (MeOH) *λ*_max_ (log *ε*): 208.0 (1.76), 308 (0.72) nm; CD (*c* 2.0 × 10^–3^ M, MeOH) *λ*max (Δ*ε*) 310 (–8.7), 254 (+4.4) nm, 217 (+4.6) nm; IR (neat) *ν*_max_ (cm^−1^): 3477, 2954, 1753, 1446, 1376, 1243, 1036. ^1^H and ^13^C NMR data, Table 1; HRESIMS *m/z* 529.2409 [M + Na]^+^ (calcd for C_27_H_38_O_9_Na, 529.2408).

### 3.4. Bioassays for Cytotoxic Activity

Cytotoxic assay was performed as previously described [23].

### 3.5. ECD Calculation

Conformational analyses for compounds **1**–**3** were performed using Maestro 10.2 in the OPLS3 molecular mechanics force-field within an energy window of 5.0 or 3.0 kcal/mol. The conformers were then further optimized with the software package Gaussian 09 at the B3LYP/6-311G(2d,p), B3LYP/6-31G(d), and B3LYP/6-311G(d,p) level for compounds **1**–**3**, respectively [24], and the harmonic vibrational frequencies were also calculated to confirm their stability. The time-dependent density functional theory (TD-DFT) methods at the CAM-B3LYP/6-31G(2d,p), B3LYP/6-31G(d), and B3LYP/6-311G(d,p) were applied to calculate the 60 lowest electronic transitions which obtained conformers in vacuum, respectively. The Gaussian function was applied to simulate the ECD spectrum of the conformers. The calculated ECD spectra were obtained according to the Boltzmann weighting of each conformer’s ECD spectrum in MeOH solution.

## 4. Conclusions

In conclusion, three new andrastin-type meroterpenoids penimeroterpenoids A–C (**1**–**3**) together with two known compounds (**4** and **5**) were isolated from the fermentation broth of the marine-derived fungi *Penicillium* sp. The structures of the new compounds were elucidated by mass spectrometry (MS), NMR, and ECD spectroscopic data. Compound **1** showed moderate cytotoxicity against A549, HCT116, and SW480 cell lines. Our findings also suggest that the fungal genus *Penicillium* is a rich source of bioactive secondary metabolites, and thus worthy of in-depth investigations.

## Figures and Tables

**Table 1:** ^1^H NMR and ^13^C NMR data (500 and 125 MHz) for **1**–**3** in CDCl_3_.

Position	1		2		3	
	*δ*_H_ (*J* in Hz)	*δ*_C_	*δ*_H_ (*J* in Hz)	*δ*_C_	*δ*_H_ (*J* in Hz)	*δ*_C_
1a	2.32, m	27.8, CH_2_	2.31, dt (12.9 5.5)	26.6 CH_2_	2.75, m	23.1, CH_2_
1b	1.01, dt (12.0 5.5)		1.01, m		2.03, m	
2	1.59, m	23.4, CH_2_	1.71, m	23.6, CH_2_	1.82, m	26.3, CH_2_
3	4.65, m	77.1, CH	4.65, t (2.5)	76.9, CH	4.73, t (2.5)	77.7, CH
4		37.0, C		37.4, C		38.7, C
5	1.78, m	47.7, CH	1.80, m	46.0, CH	2.45, dd (11.1, 6.8)	41.8, CH
6a	2.02, m	16.9, CH_2_	2.16, m	16.9, CH_2_	1.62, m	18.2, CH_2_
6b	1.81, m		1.81, m		1.62, m	
7a	2.81, td (13.5, 3.5)	30.8, CH_2_	2.84, td (13.1, 4.4)	32.6, CH_2_	2.72, m	29.7, CH_2_
7b	2.36, m		2.09, m		1.95, m	
8		38.6, C		39.7, C		39.3, C
9	2.19, s	53.5, CH	2.19, s	147.9, C		132.7, C
10		52.3, C		55.1, C		141.9, C
11	5.82, s	126.4, CH	5.55, s	125.8, CH	4.75, s	70.8, CH
12		132.9, C		76.0, C		79.4, C
13		60.9, C		53.1, C		55.0, C
14		70.6, C		71.8, C		72.7, C
15		210.6, C		202.0, C		197.7, C
16		72.1, C		75.6, C		77.0, C
17		206.8, C		202.3, C		204.4, C
18	1.38, s	19.6, CH_3_	1.31, s	7.6, CH_3_	1.26, s	7.4, CH_3_
19	1.29, s	16.4, CH_3_	1.25, s	10.4, CH_3_	1.42, s	10.8, CH_3_
20	1.68, s	18.9, CH_3_	1.26, s	24.4, CH_3_	1.36, s	22.2, CH_3_
21	10.1, s	204.5, CH	10.1, s	202.1, CH		170.8, CH
22		170.7, C		170.9, C	2.12, s	21.3, CH_3_
23	2.10, s	21.3, CH_3_	2.10, s	21.6, CH_3_	0.87, s	21.3, CH_3_
24	0.88, s	21.4, CH_3_	0.93, s	21.4, CH_3_	1.00, s	24.8, CH_3_
25	0.94, s	26.5, CH_3_	0.96, s	26.8, CH_3_	1.75, s	24.8, C
26	1.15, s	19.9, CH_3_	1.44, s	26.2, CH_3_		168.0, C
27		167.3, C		167.5, C	3.61, s	52.0, CH_3_
28	3.61, s	52.0, CH_3_	3.62, s	52.3, CH_3_		

## Data Availability

Data is contained within the article or supplementary material.

## Supplementary Materials

The following are available online at https://www.mdpi.com/article/10.3390/md19040189/s1, The 1D and 2D NMR spectra of the new compounds **1**–**3** are supplied.
