# Supplementary material for: New Andrastin-Type Meroterpenoids from the Marine-Derived Fungus Penicillium sp
Source: Mar Drugs. 2021 Mar 27;19(4):189. doi: 10.3390/md19040189 (PMC8066695; doi:10.3390/md19040189)
Supplement: Supplementary file 1 [file marinedrugs-19-00189-s001.pdf]

Supplemental material

New Andrastin-type Meroterpenoids from the Marine-Derived Fungus *Penicillium* sp.

Jinwei Ren [1,2,†], Ruiyun Huo [1,2,†], Gaoran Liu [1,2], and Ling Liu [1,2,*]

1 State Key Laboratory of Mycology, Institute of Microbiology, Chinese Academy of Sciences, Beijing 100101, China; renjw@im.ac.cn (J.R.), ruiyunhuo@163.com (R.H.), liugaoran@163.com (G.L.)

2 University of Chinese Academy of Sciences, Beijing 100039, China

* Correspondence: liul@im.ac.cn (L.L.),Tel.: +86-10-64806153

† These authors contributed equally to this work.

Table of Contents

Figure S1. ^{1}H NMR spectrum of penimeroterpenoid A (**1**; 500 MHz, $CDCl_3$) S2

Figure S2. ^{13}C NMR spectrum of penimeroterpenoid A (**1**; 125 MHz, $CDCl_3$) S3

Figure S3. ^{1}H-^{1}H COSY spectrum of penimeroterpenoid A (**1**; 500 MHz, $CDCl_3$) S4

Figure S4. HSQC spectrum of penimeroterpenoid A (**1**; 500 MHz, $CDCl_3$) S5

Figure S5. HMBC spectrum of penimeroterpenoid A (**1**; 500 MHz, $CDCl_3$) S6

Figure S6. NOESY spectrum of penimeroterpenoid A (**1**; 500 MHz, $CDCl_3$) S7

Figure S7. ^{1}H NMR spectrum of penimeroterpenoid B (**2**; 500 MHz, $CDCl_3$) S8

Figure S8. ^{13}C NMR spectrum of penimeroterpenoid B (**2**; 125 MHz, $CDCl_3$) S9

Figure S9. ^{1}H-^{1}H COSY spectrum of penimeroterpenoid B (**2**; 500 MHz, $CDCl_3$) S10

Figure S10. HSQC spectrum of penimeroterpenoid B (**2**; 500 MHz, $CDCl_3$) S11

Figure S11. HMBC spectrum of penimeroterpenoid B (**2**; 500 MHz, $CDCl_3$) S12

Figure S12. NOESY spectrum of penimeroterpenoid B (**2**; 500 MHz, $CDCl_3$) S13

Figure S13. ^{1}H NMR spectrum of penimeroterpenoid C (**3**; 500 MHz, $CDCl_3$) S13

Figure S14. ^{13}C NMR spectrum of penimeroterpenoid C (**3**; 125 MHz, $CDCl_3$) S15

Figure S15. ^{1}H-^{1}H COSY spectrum of penimeroterpenoid C (**3**; 500 MHz, $CDCl_3$) S16

Figure S16. HSQC spectrum of penimeroterpenoid C (**3**; 500 MHz, $CDCl_3$) S17

Figure S17. HMBC spectrum of penimeroterpenoid C (**3**; 400 MHz, $CDCl_3$) S18

Figure S18. NOESY spectrum of penimeroterpenoid C (**3**; 500 MHz, $CDCl_3$) S19

Figure S19. ^{1}H NMR spectrum of andrastone E (**4**; 500 MHz, $CDCl_3$) S20

Figure S20. ^{13}C NMR spectrum of andrastone E (**4**; 125 MHz, $CDCl_3$) S21

Figure S21. NOESY spectrum of andrastone E (**4**; 500 MHz, $CDCl_3$) S22

Figure S22. ECD conformers of penimeroterpenoids A–C (**1**–**3**) S23

***** Author to whom correspondence should be addressed; E-mail: liul@im.ac.cn; Tel: 86-10-64806153.

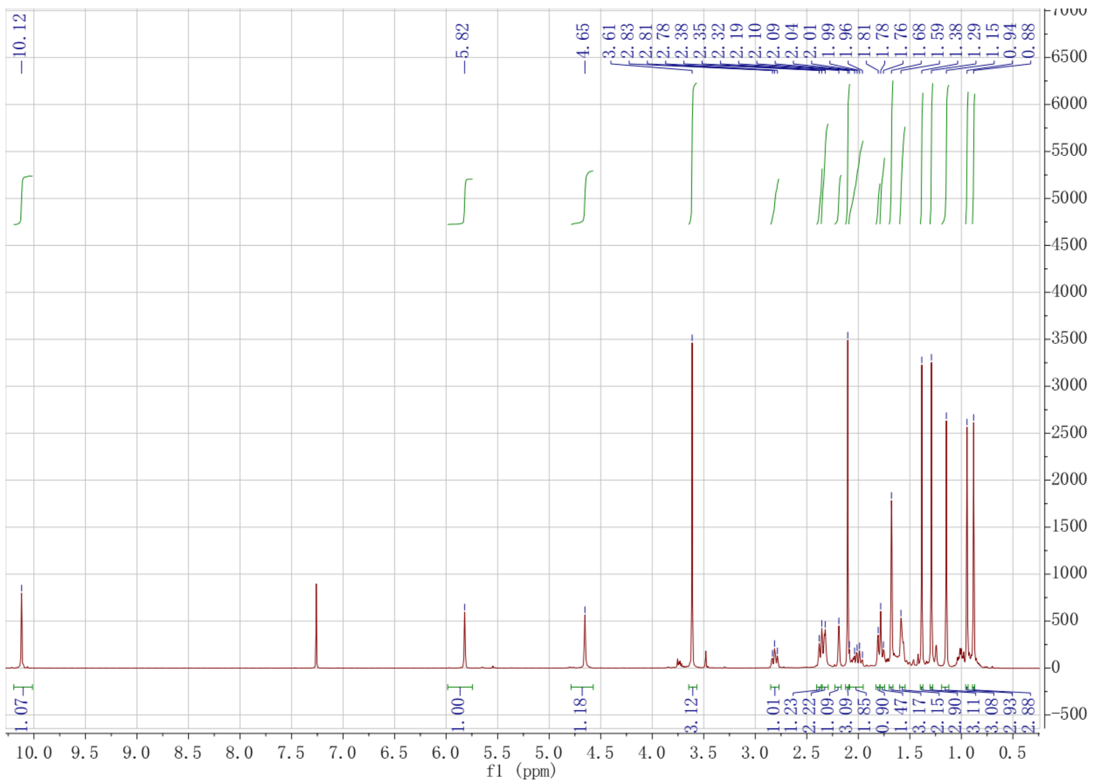

Figure S1. ^{1}H NMR spectrum of penimeroterpenoid A (**1**; 500 MHz, $CDCl_3$)

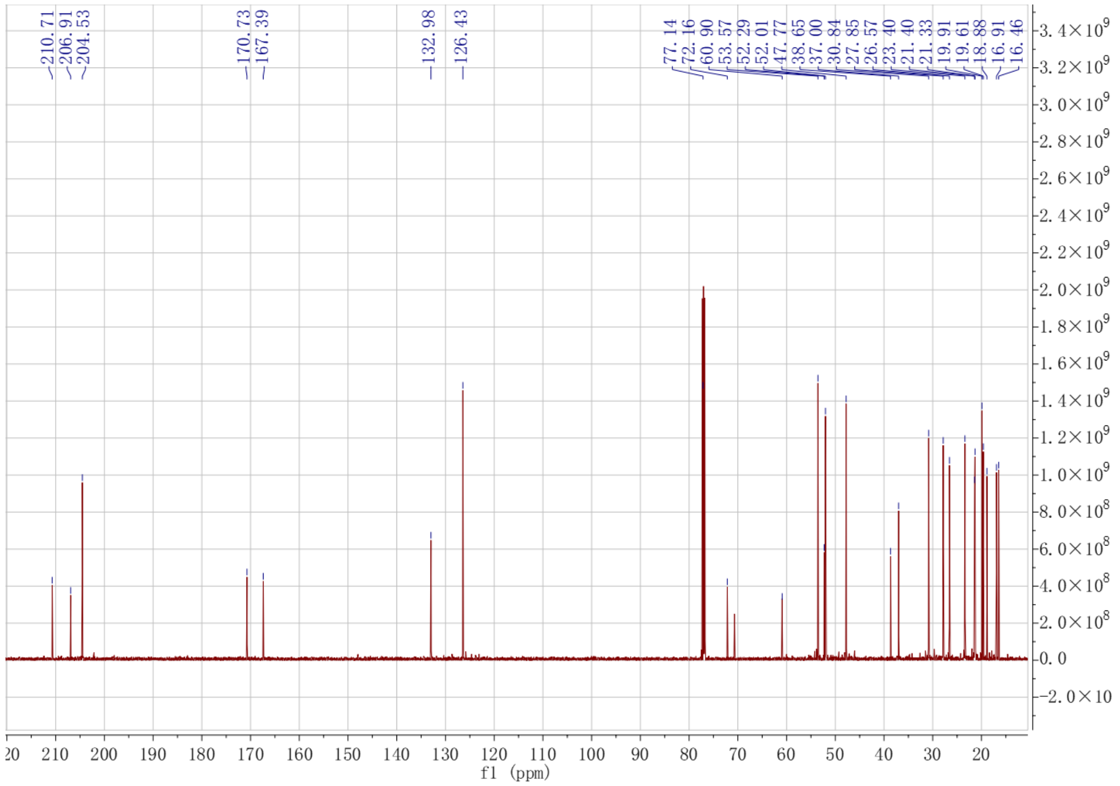

Figure S2. ^{13}C NMR spectrum of penimeroterpenoid A (**1**; 125 MHz, $CDCl_3$)

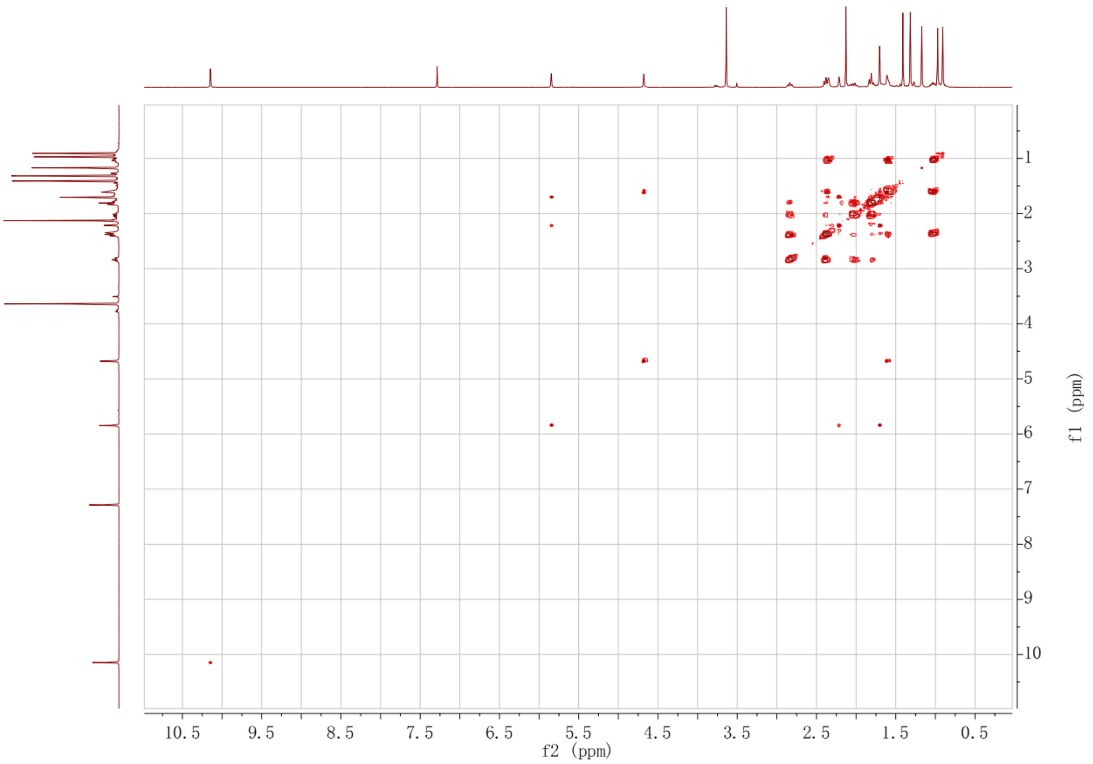

Figure S3. ^{1}H-^{1}H COSY spectrum of penimeroterpenoid A (**1**; 500 MHz, $CDCl_3$)

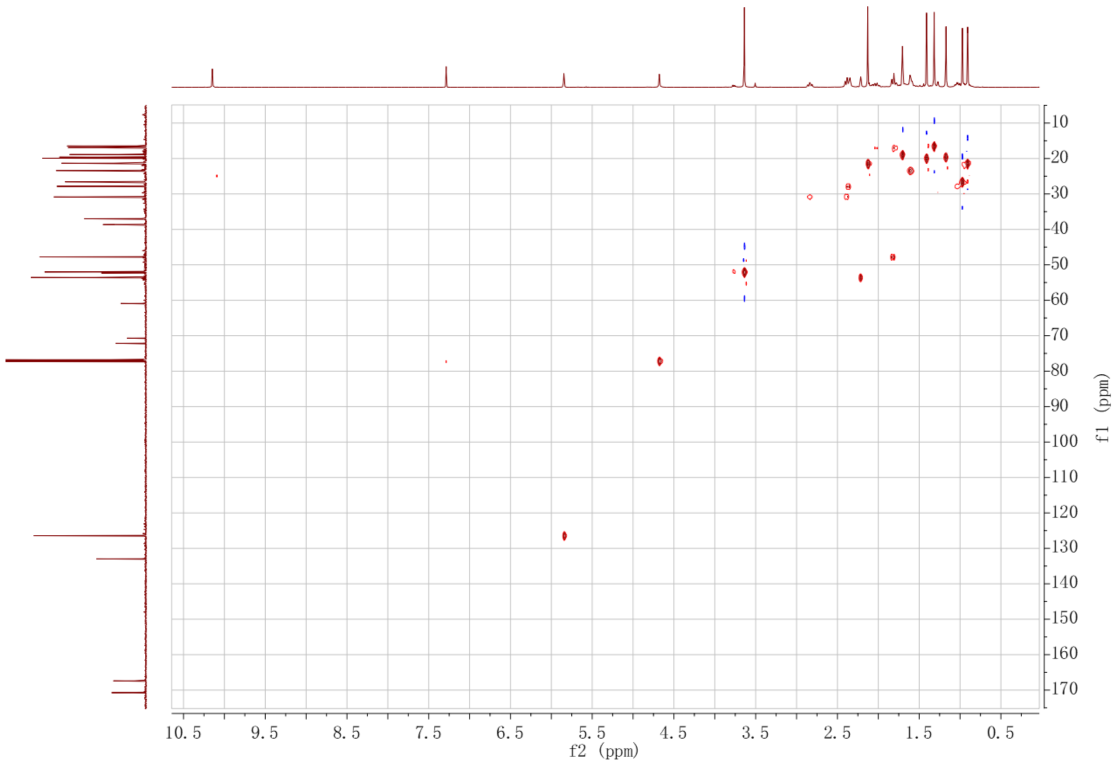

Figure S4. HSQC spectrum of penimeroterpenoid A (**1**; 500 MHz, $CDCl_3$)

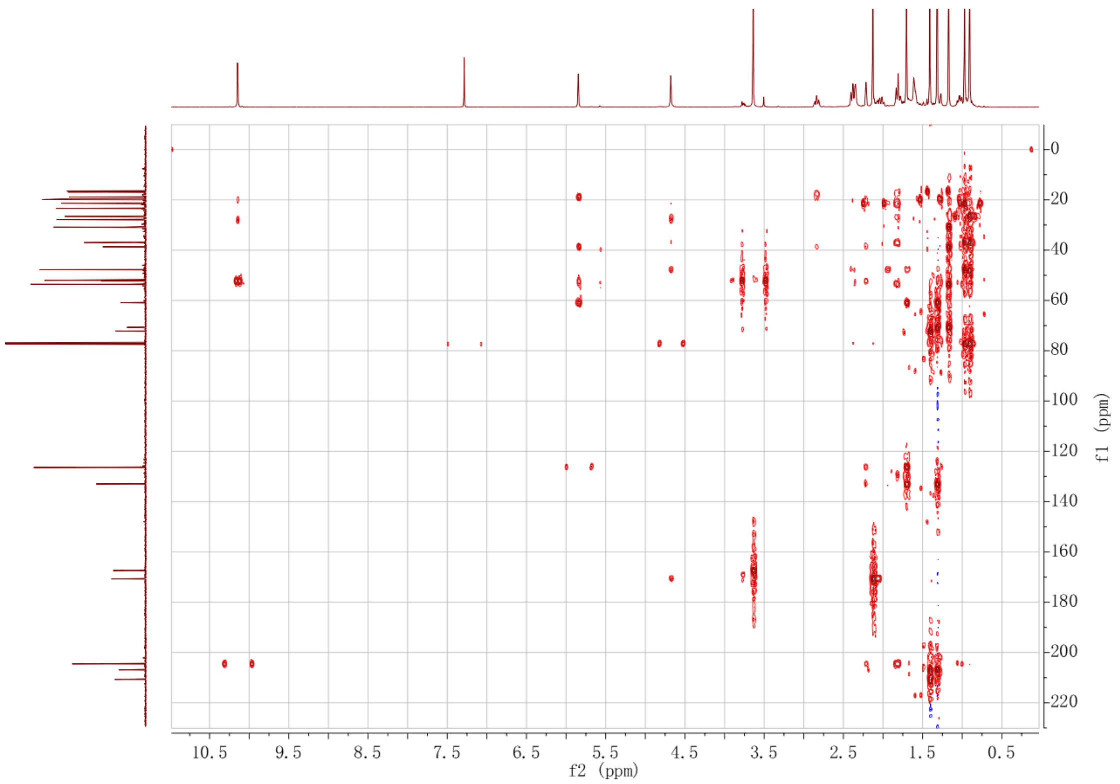

Figure S5. HMBC spectrum of penimeroterpenoid A (**1**; 500 MHz, $CDCl_3$)

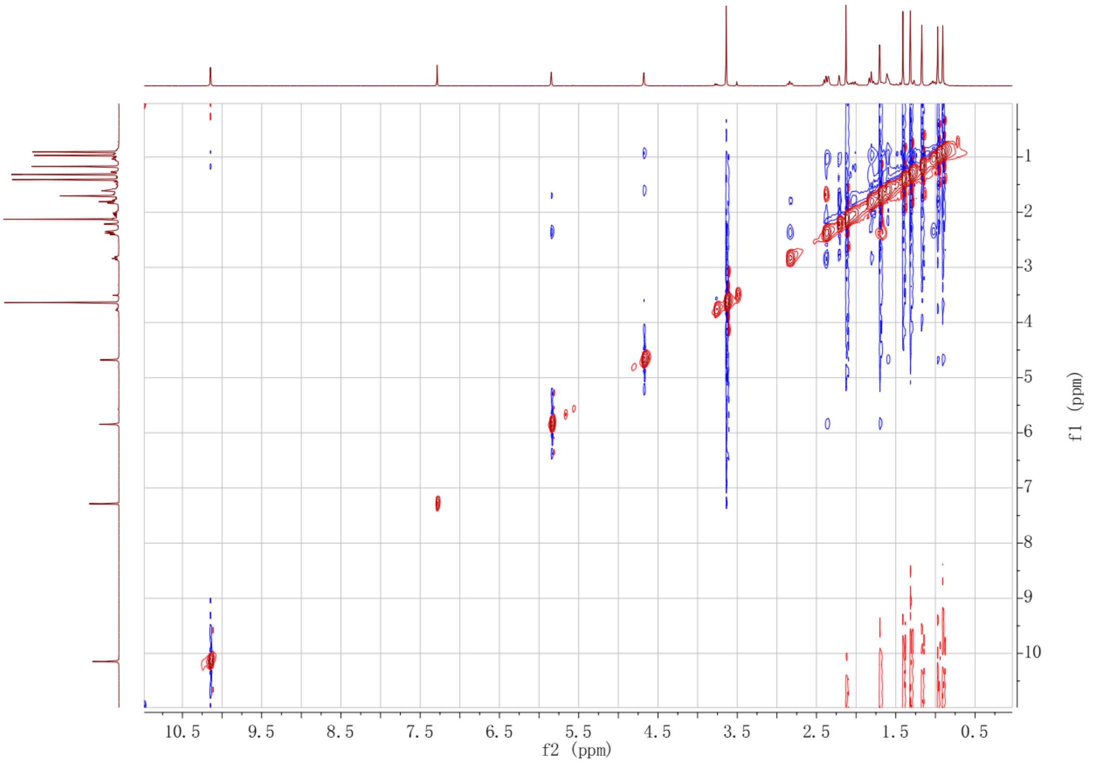

Figure S6. NOESY spectrum of penimeroterpenoid A (**1**; 500 MHz, $CDCl_3$)

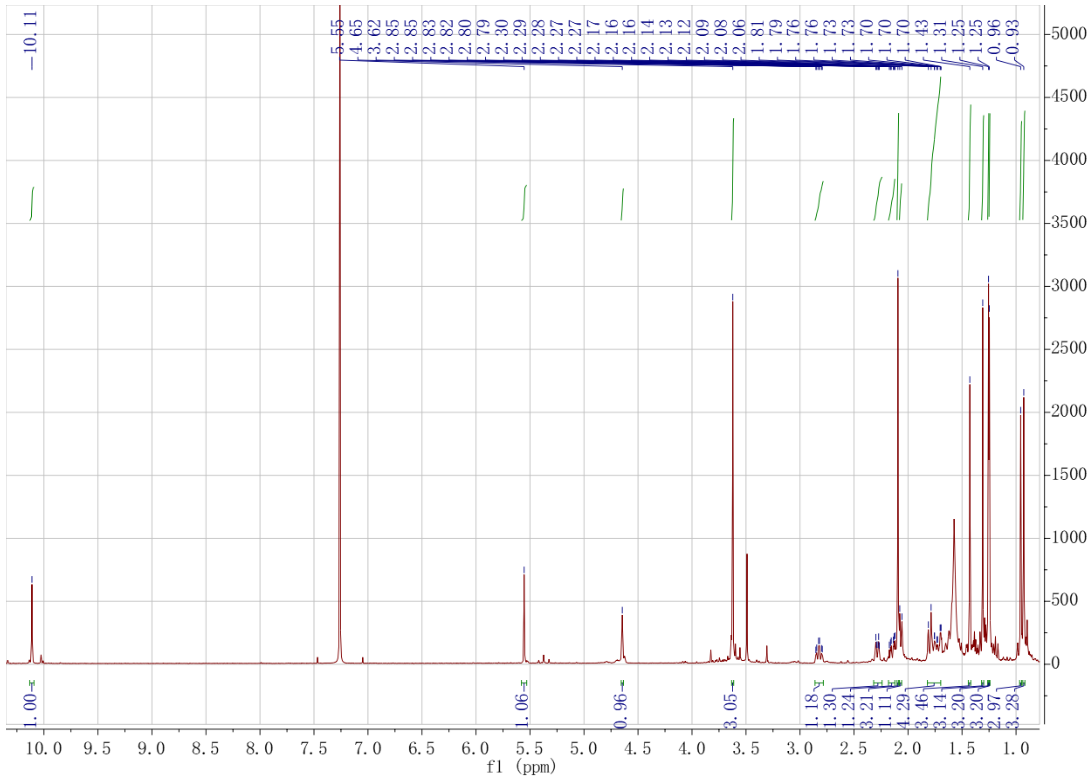

Figure S7. ^{1}H NMR spectrum of penimeroterpenoid B (**2**; 500 MHz, $CDCl_3$)

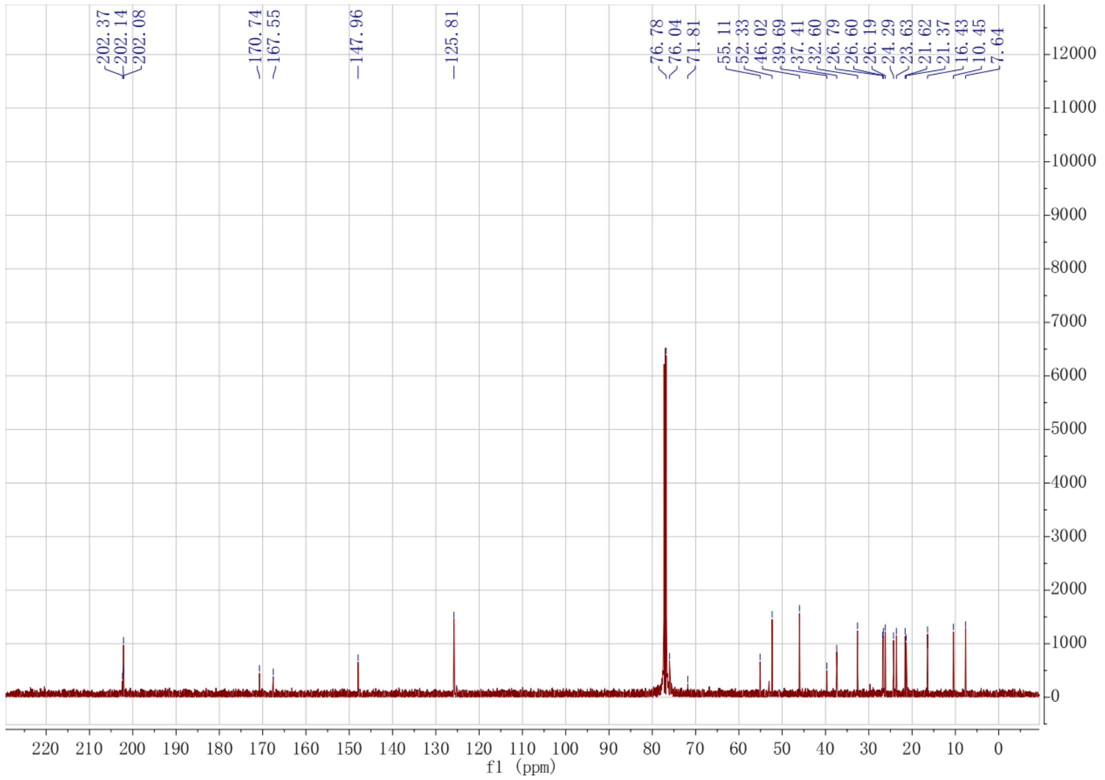

Figure S8. ^{13}C NMR spectrum of penimeroterpenoid B (**2**; 125 MHz, $CDCl_3$)

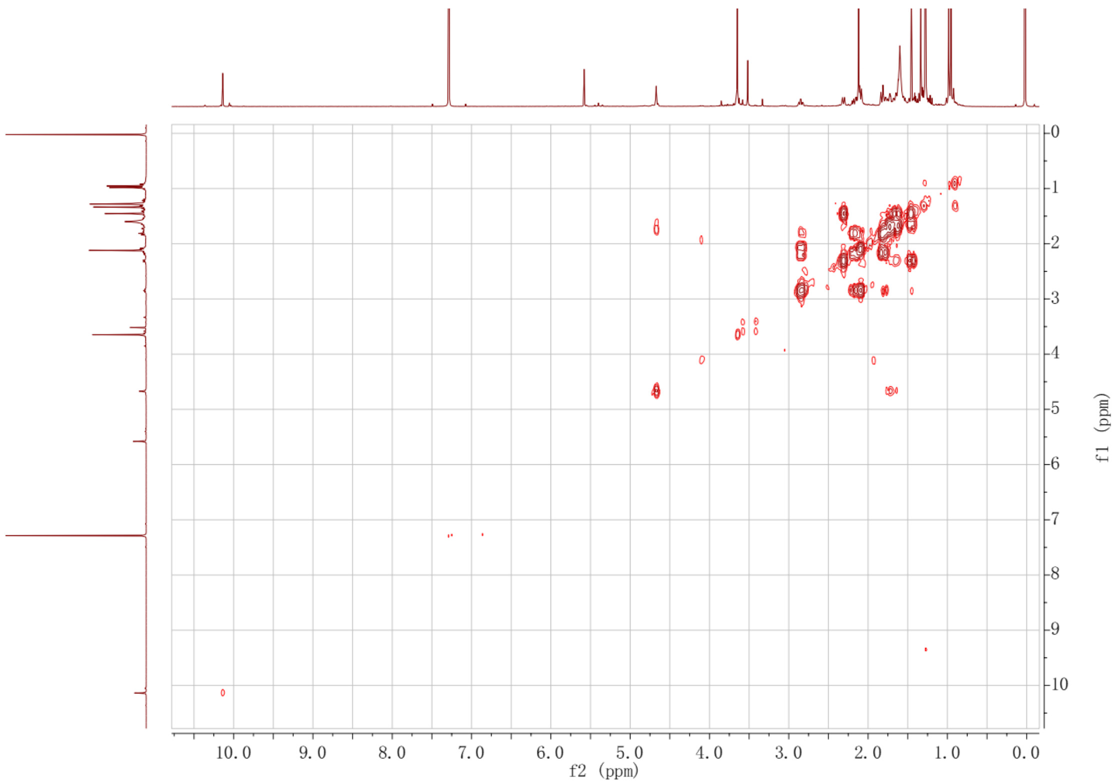

Figure S9. ^1H-^1H COSY spectrum of penimeroterpenoid B (**2**; 500 MHz, $CDCl_3$)

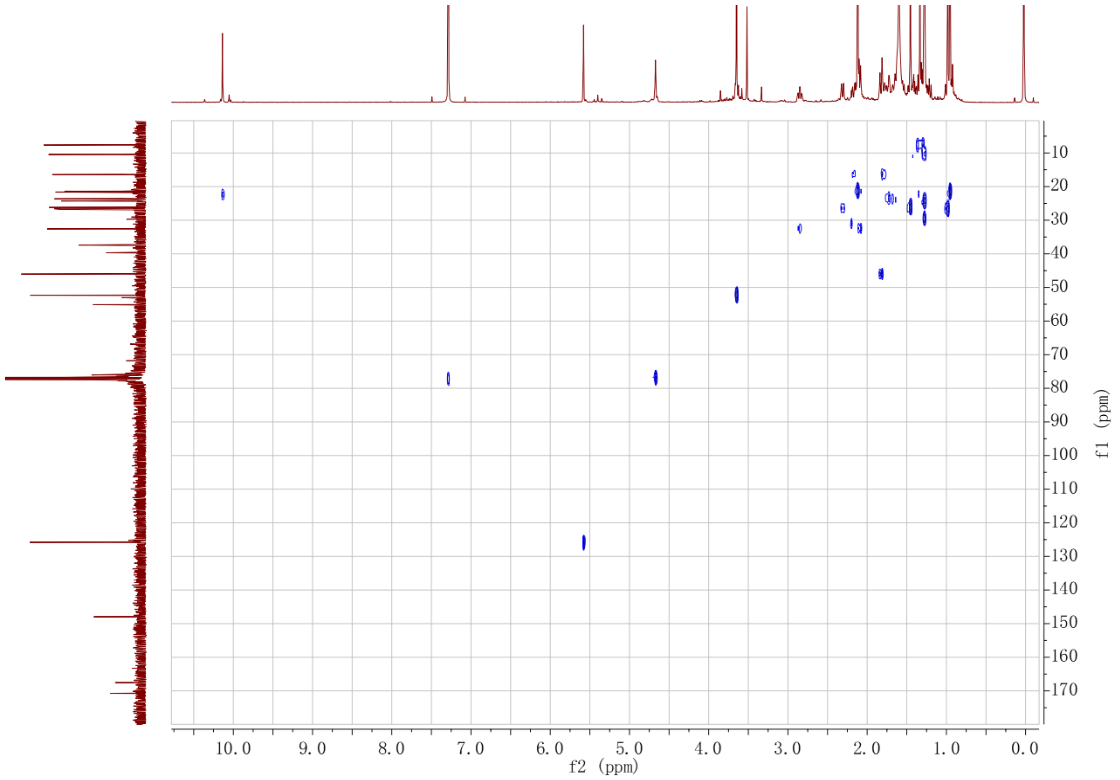

Figure S10. HSQC spectrum of penimeroterpenoid B (**2**; 500 MHz, $CDCl_3$)

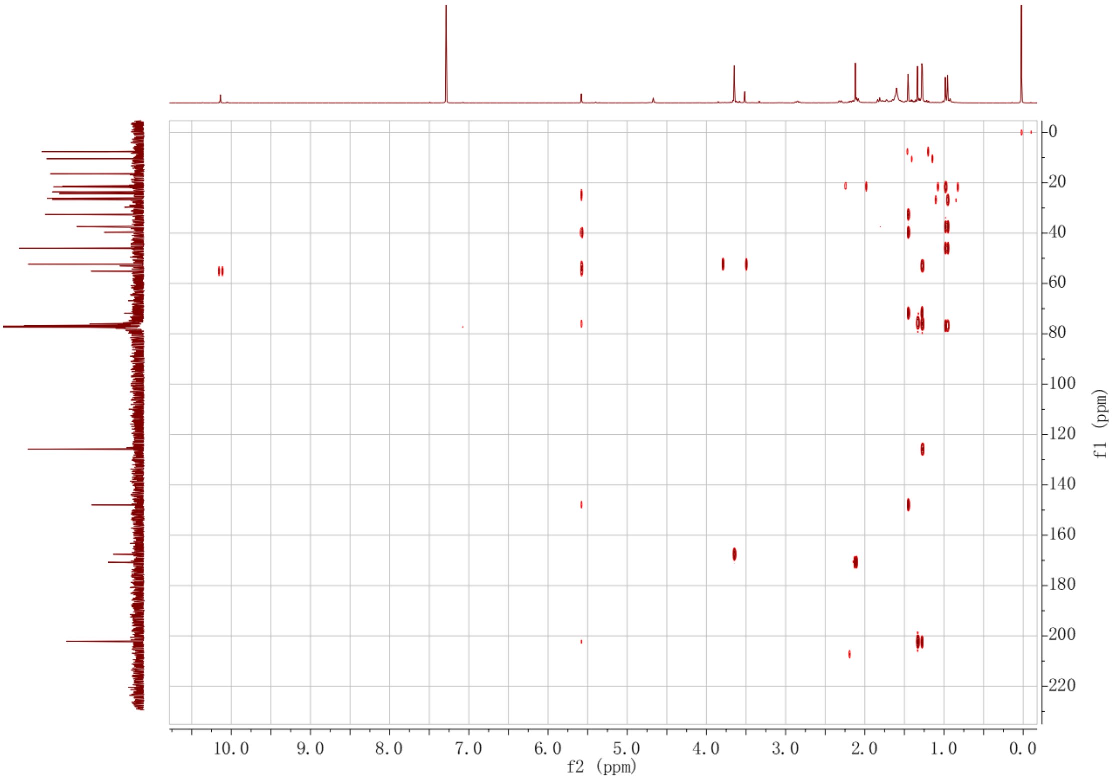

Figure S11. HMBC spectrum of penimeroterpenoid B (**2**; 500 MHz, $CDCl_3$)

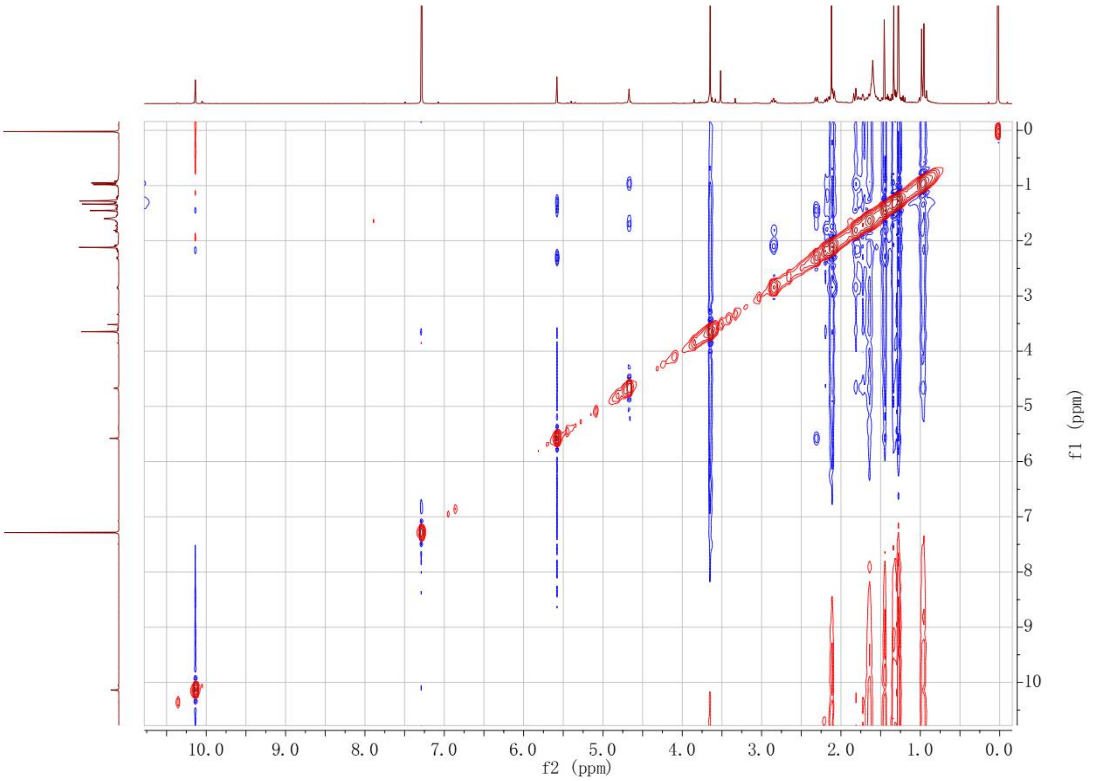

Figure S12. NOESY spectrum of penimeroterpenoid B (**2**; 500 MHz, $CDCl_3$)

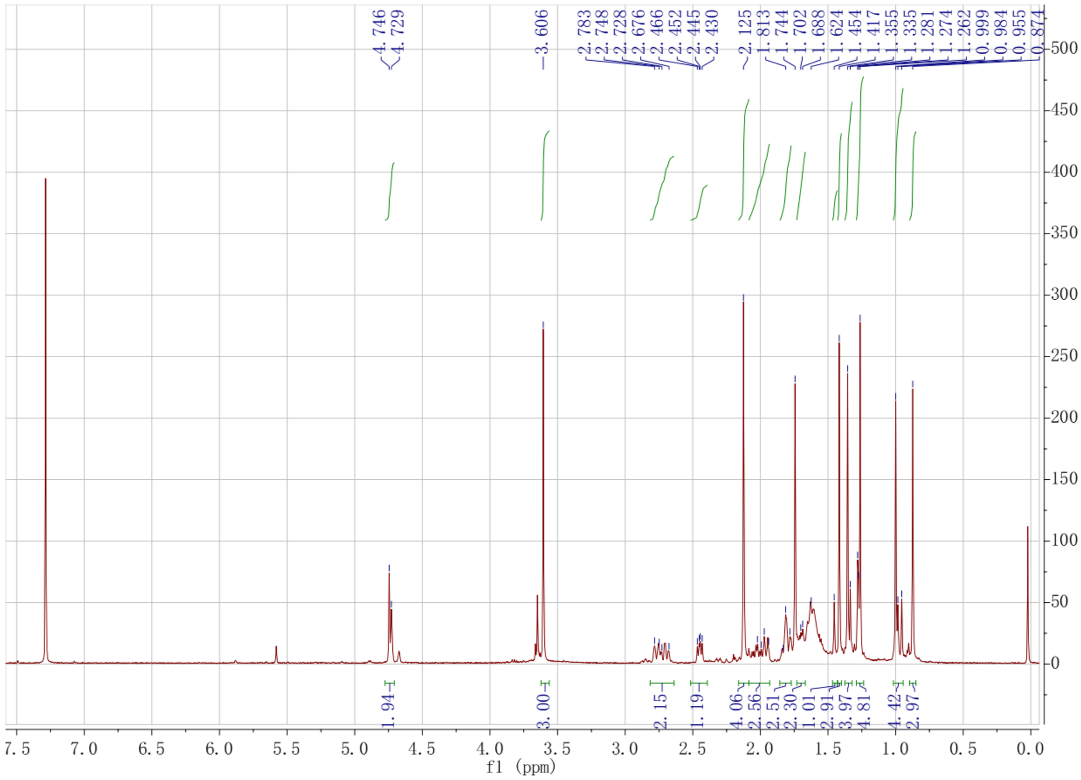

Figure S13. ^{1}H NMR spectrum of penimeroterpenoid C (**3**; 500 MHz, $CDCl_3$)

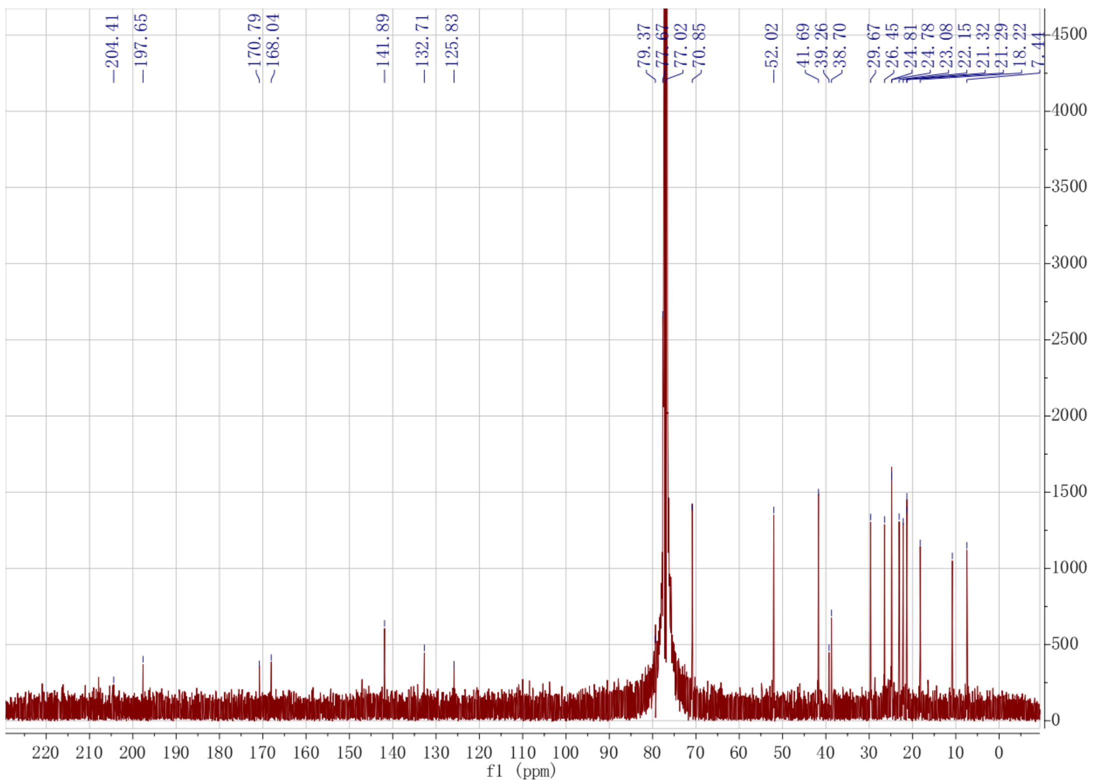

Figure S14. ^{13}C NMR spectrum of penimeroterpenoid C (**3**; 125 MHz, $CDCl_3$)

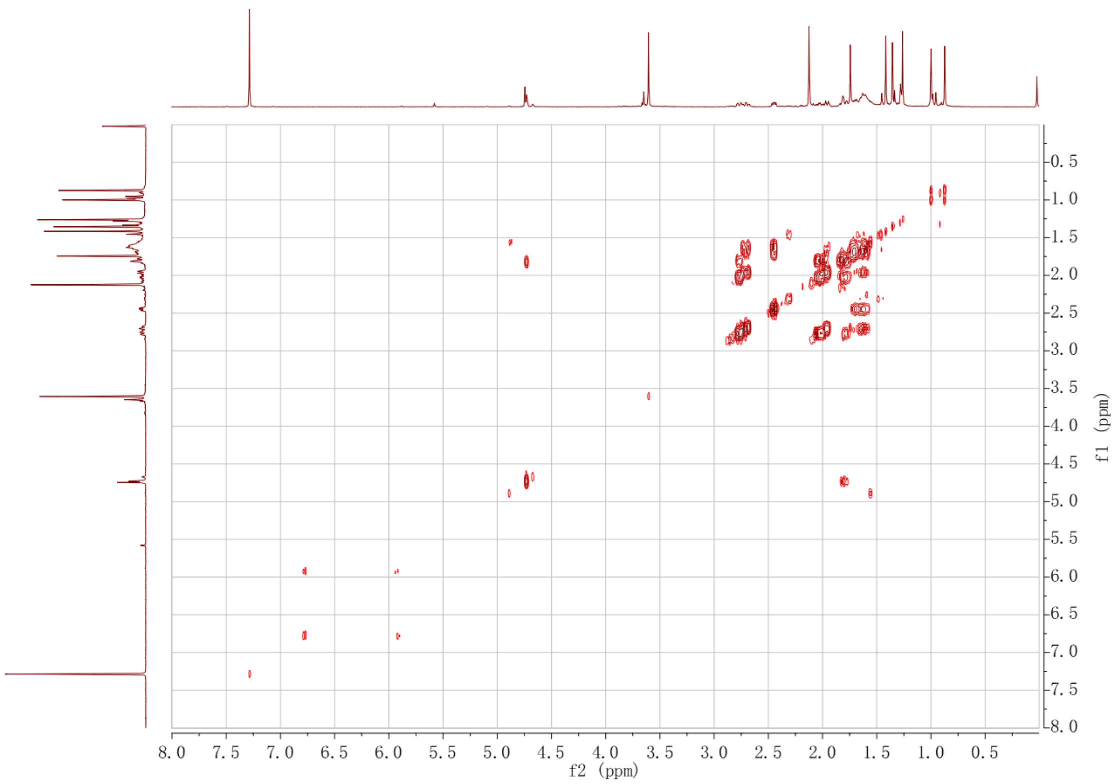

Figure S15. ^{1}H-^{1}H COSY spectrum of penimeroterpenoid C (**3**; 500 MHz, $CDCl_3$)

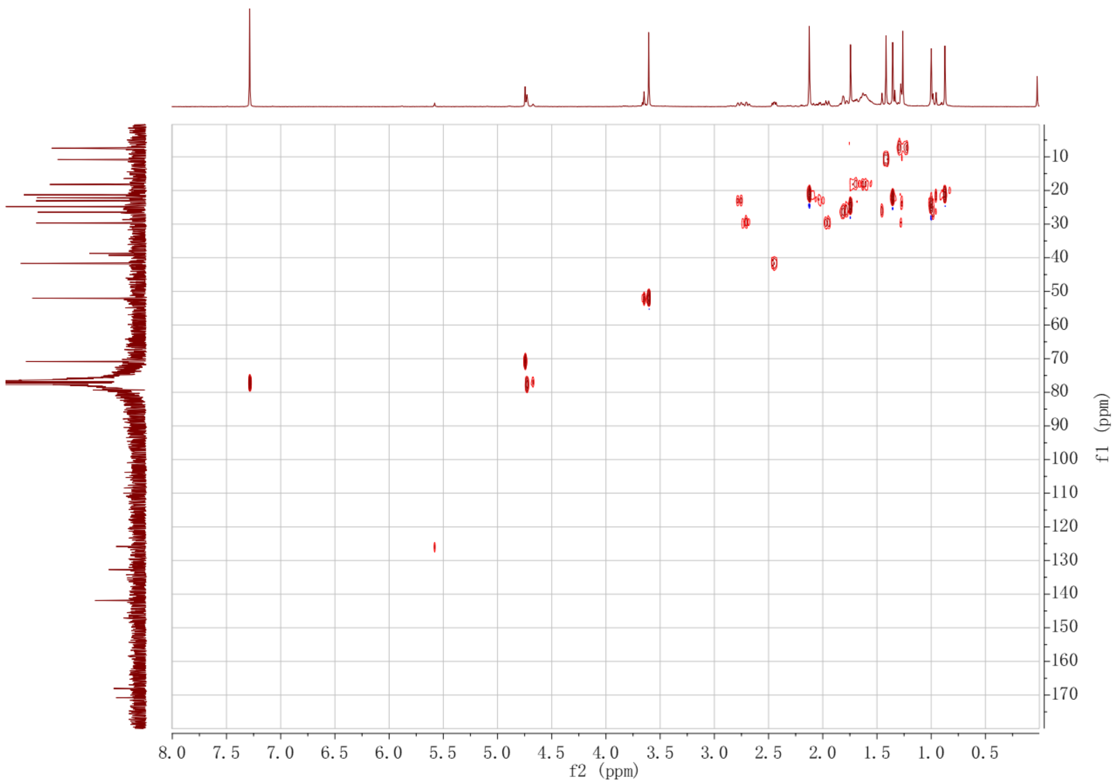

Figure S16. HSQC spectrum of penimeroterpenoid C (**3**; 500 MHz, $CDCl_3$)

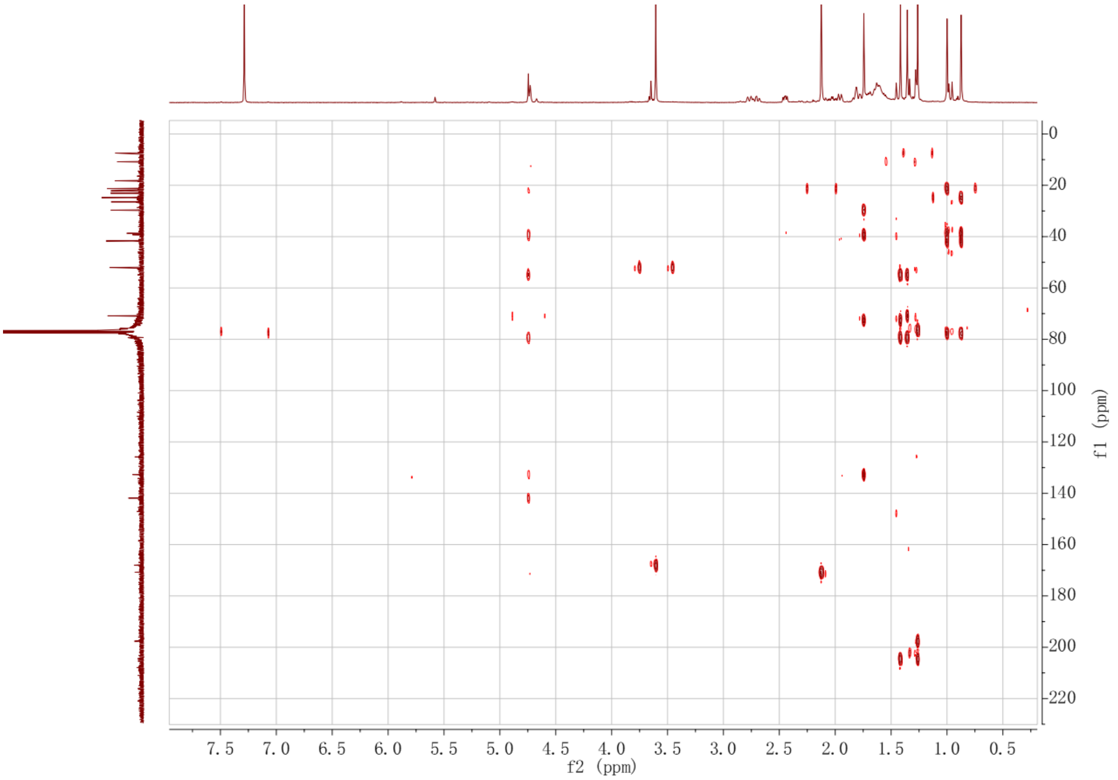

Figure S17. HMBC spectrum of penimeroterpenoid C (**3**; 500 MHz, $CDCl_3$)

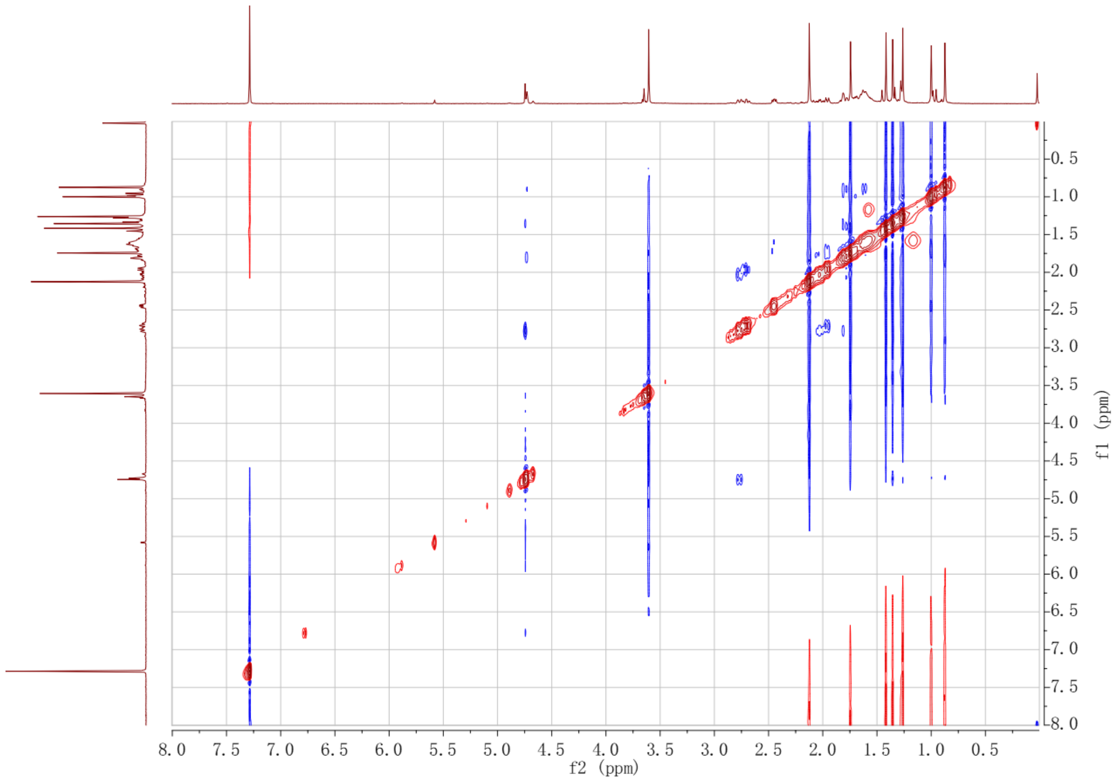

Figure S18. NOESY spectrum of penimeroterpenoid C (**3**; 500 MHz, $CDCl_3$)

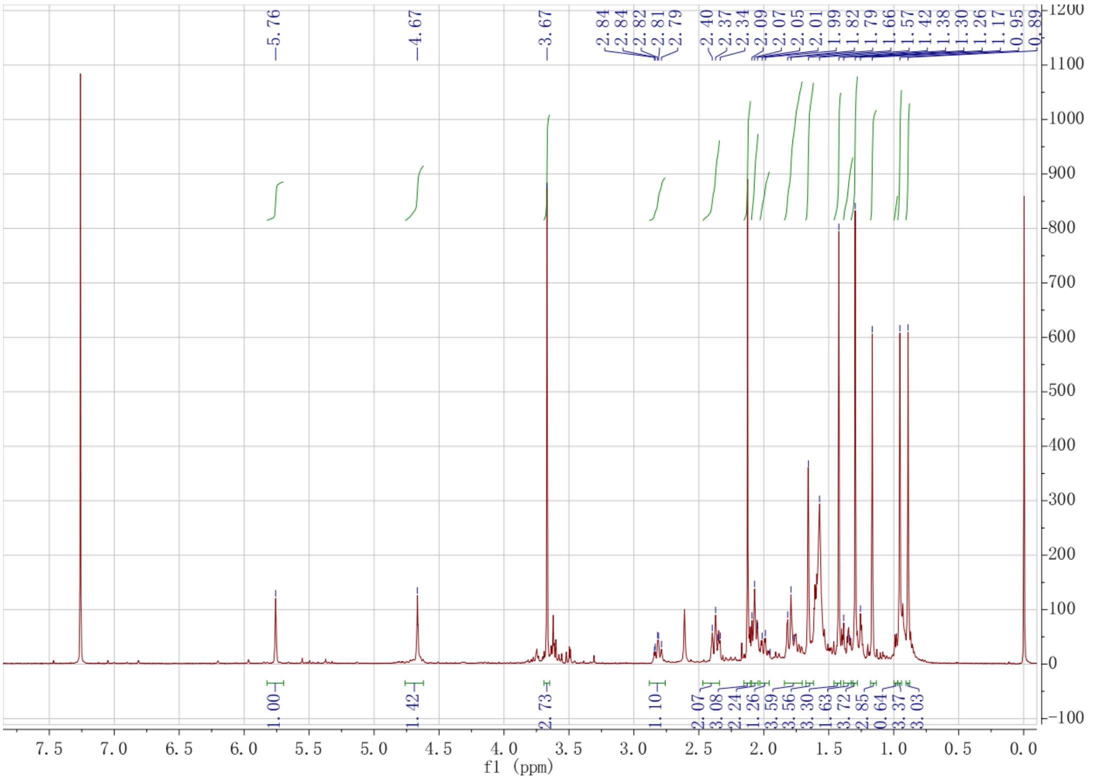

Figure S19. ^{1}H NMR spectrum of andrastone E (**4**; 500 MHz, $CDCl_3$)

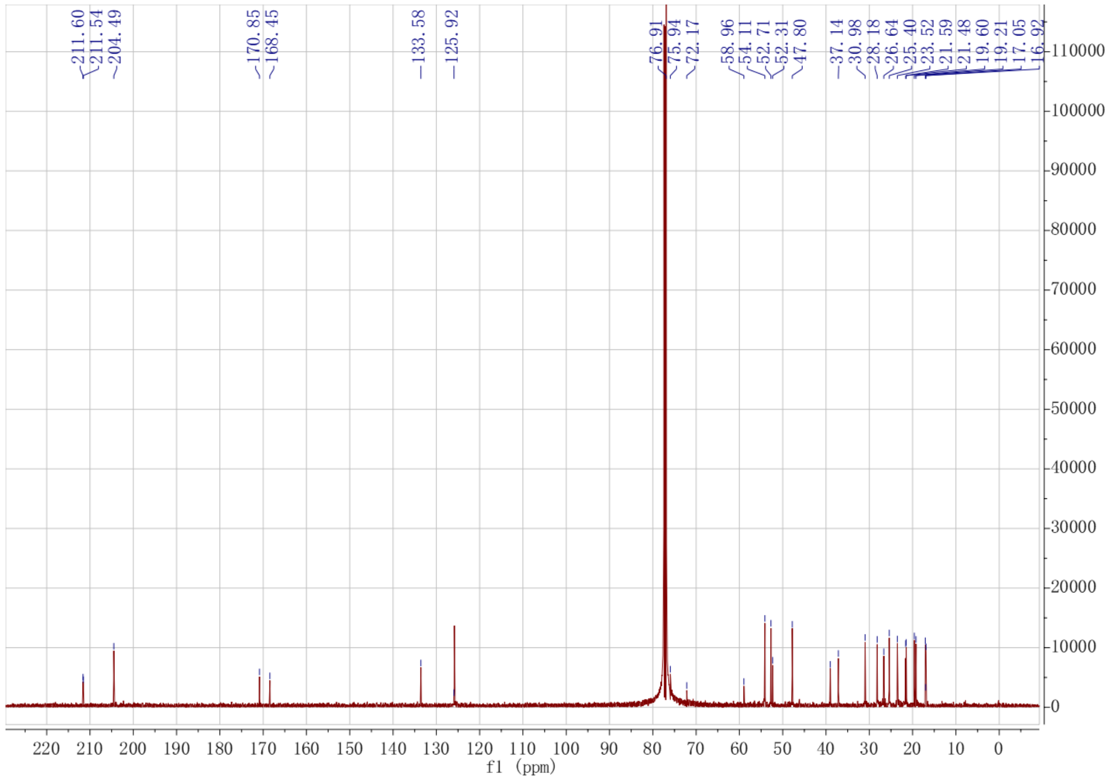

Figure S20. ^{13}C NMR spectrum of andrastone E (**4**; 125 MHz, $CDCl_3$)

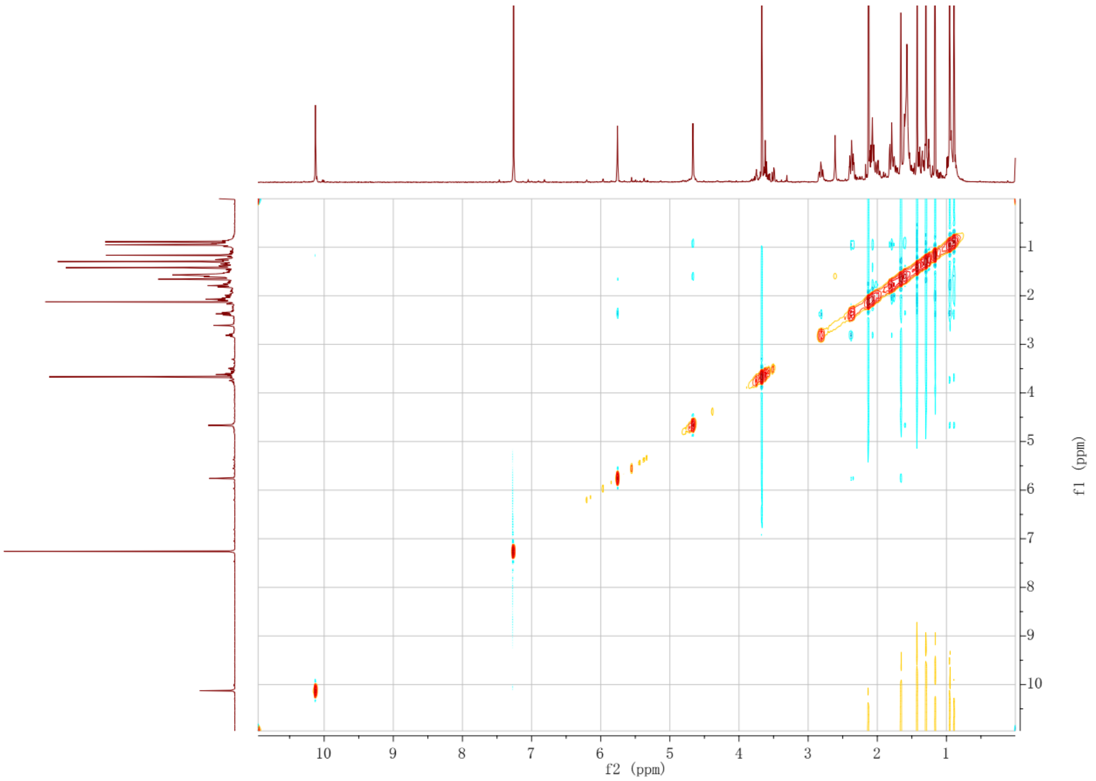

Figure S21. NOESY NMR spectrum of andrastone E (**4**; 500 MHz, $CDCl_3$)

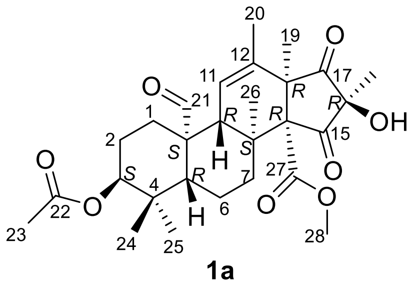

1a

Conformers	Populations
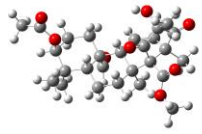	4.15 %
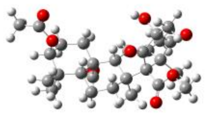	37.49 %
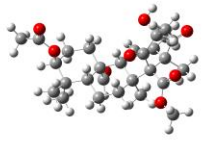	1.23 %
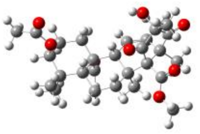	4.05 %

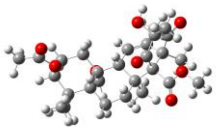	10.71 %
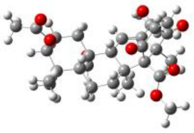	1.82 %
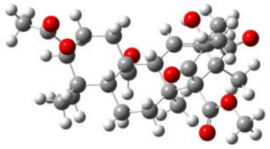	35.27 %
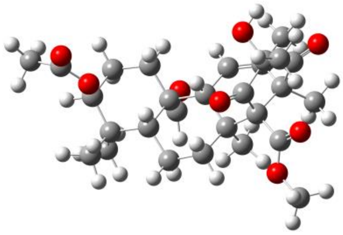	1.20 %
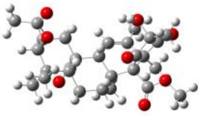	4.07 %

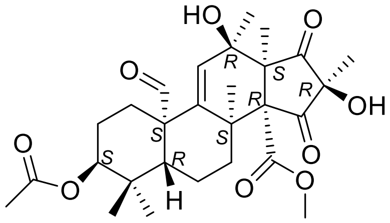 **2a**	
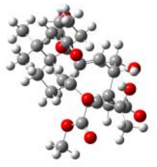	7.71 %
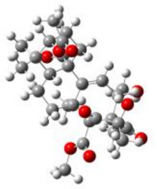	8.98 %
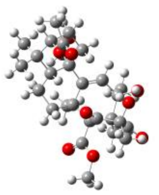	45.92 %
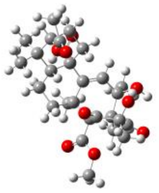	3.51 %

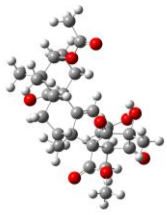	30.35 %
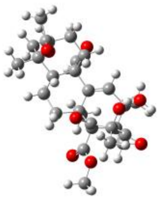	3.53 %
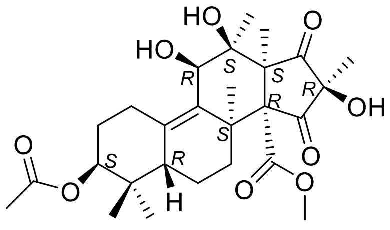 **3a**	
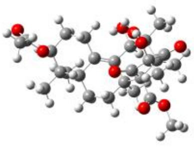	8.33 %
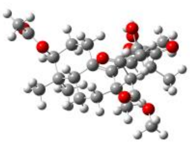	8.24 %

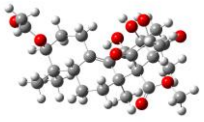	8.42 %
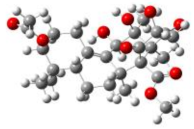	29.38 %
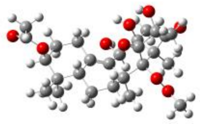	4.03 %
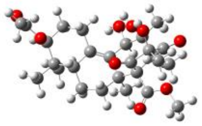	8.00 %
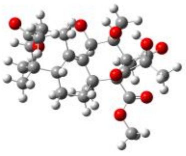	29.61 %

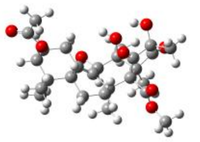	3.99 %

Figure S22. ECD conformers of penimeroterpenoids A–C (**1**–**3**)
